# Mucosal delivery of *Lactococcus lactis* carrying an anti-TNF scFv expression vector ameliorates experimental colitis in mice

**DOI:** 10.1186/s12896-019-0518-6

**Published:** 2019-06-25

**Authors:** Maria José Chiabai, Juliana Franco Almeida, Mariana Gabriela Dantas de Azevedo, Suelen Soares Fernandes, Vanessa Bastos Pereira, Raffael Júnio Araújo de Castro, Márcio Sousa Jerônimo, Isabel Garcia Sousa, Leonora Maciel de Souza Vianna, Anderson Miyoshi, Anamelia Lorenzetti Bocca, Andrea Queiroz Maranhão, Marcelo Macedo Brigido

**Affiliations:** 1 Laboratório de Imunologia Molecular, Departamento de Biologia Molecular, Universidade de Brasília, Brasília, Distrito Federal Brazil; 2 Centro de Biotecnologia, Departamento de Biologia Celular e Molecular, Universidade Federal da Paraíba, João Pessoa, Paraíba Brazil; 3 Laboratório de Tecnologia Genética, Departamento de Biologia Geral, Universidade Federal de Minas Gerais, Belo Horizonte, Minas Gerais Brazil; 4 Laboratório de Imunologia Aplicada, Departamento de Biologia Celular, Universidade de Brasília, Brasília, Distrito Federal Brazil; 5 Departmento de Patologia, Escola de Medicina, Universidade de Brasília, Brasília, Distrito Federal Brazil; 6 Instituto Nacional de Investigação em Imunologia, INCTii, Brasília, Distrito Federal Brazil

**Keywords:** *Lactococcus lactis*, Mucosal delivery, Anti-TNFα, scFv, Colitis, DSS

## Abstract

**Background:**

Anti-Tumor Necrosis Factor-alpha therapy has become clinically important for treating inflammatory bowel disease. However, the use of conventional immunotherapy requires a systemic exposure of patients and collateral side effects. Lactic acid bacteria have been shown to be effective as mucosal delivering system for cytokine and single domain antibodies, and it is amenable to clinical purposes. Therefore, lactic acid bacteria may function as vehicles for delivery of therapeutic antibodies molecules to the gastrointestinal tract restricting the pharmacological effect towards the gut. Here, we use the mucosal delivery of *Lactococcus lactis* carrying an *anti-TNFα* scFv expression plasmid on a DSS-induced colitis model in mice.

**Results:**

Experimental colitis was induced with DSS administered in drinking water. *L. lactis* carrying the scFv expression vector was introduced by gavage. After four days of treatment, animals showed a significant improvement in histological score and disease activity index compared to those of untreated animals. Moreover, treated mice display IL-6, IL17A, IL1β, IL10 and FOXP3 mRNA levels similar to health control mice. Therefore, morphological and molecular markers suggest amelioration of the experimentally induced colitis.

**Conclusion:**

These results provide evidence for the use of this alternative system for delivering therapeutic biopharmaceuticals in loco for treating inflammatory bowel disease, paving the way for a novel low-cost and site-specific biotechnological route for the treatment of inflammatory disorders.

**Electronic supplementary material:**

The online version of this article (10.1186/s12896-019-0518-6) contains supplementary material, which is available to authorized users.

## Background

Inflammatory bowel disease (IBD), which includes Crohn’s disease (CD) and ulcerative colitis (UC), is characterized by chronic inflammation of the gastrointestinal tract (GIT) and a cryptogenic origin [1], with a global incidence of 0.3% [2]. The characteristic tissue damage of the disorder occurs due to the abnormal expression of anti-inflammatory and pro-inflammatory molecules from both the innate and adaptive responses [3]. Tumor necrosis factor-α (TNFα) plays a crucial role in the pathogenesis of IBD, and indeed, monoclonal antibodies targeting TNFα are the most powerful treatment for IBD; however, the intravenous administration route causes immunogenic and systemic side effects [4, 5]. Therefore, the local delivery of this pharmaceutical would benefit patients restricting therapy towards the inflamed tissue [6].

Recently, using bacteria as a vehicle, novel approaches for the treatment of intestinal inflammation in IBD animal models have been proposed, showing promising anti-inflammatory results such as those described by Gomes-Santos et al. [7] and Luerce et al. [8]. The *Lactococcus lactis* subspecies cremoris MG1363 is one of the most explored bacteria; it is a noninvasive and nonpathogenic gram-positive species. It is the best characterized microorganism of the group named lactic acid bacteria (LAB) and is generally regarded as safe (GRAS) by the U. S. Food and Drug Administration (FDA) [9, 10]. Although these bacteria are used in the manufacture of dairy products such as cheese and yogurt [11], *L. lactis* subsp. cremoris MG1363 is considered a potential strategy for the treatment of IBD, once it has the ability to survive the gastric acid environment and is able to replicate and deliver therapeutic molecules locally to the GIT [12]. Moreover, the medical use of engineered bacteria to produce biopharmaceuticals will pave the way for a novel biotechnological route for the low-cost treatment of immune disorders.

The use of LAB as a drug delivery system has been proposed [9] as a substitute for the oral administration of biopharmaceuticals [6, 13, 14]. However, bacterial expression systems show a limited capacity for recombinant protein production. Complex heterologous eukaryotic protein production in bacteria is normally limited by the lack of specific chaperones and other modification enzymes. Therefore, the efficient delivery of monoclonal antibodies to the animal gut depends on novel strategies. In this work, we explored the ability of *L. lactis* MG1363 FnBPA+ [15] to locally deliver a single-chain fragment variable (scFv) of anti-TNFα antibody cloned in the eukaryotic expression plasmid pValac [16] for expression in the GIT lining. We use this delivery system in a dextran sulfate sodium (DSS)-induced colitis in mice and tested its effect on the inflammatory process. We showed that treating mice orally with *L. lactis* carrying pValac::*anti-TNFα* ameliorates disease indexes as well as immunological and molecular markers. The data support the use of this alternative delivery system for treating IBD.

## Results

### Construction of pValac::*anti-TNFα*

The synthetic *anti-TNFα* coding ORF was cloned into the pValac vector (Additional file 1: Figure S1A), forming an expression cassette for eukaryotic cells. The pValac::*anti-TNFα* construction was checked by restriction endonuclease digestion profile, PCR and sequencing to confirm ORF integrity (data not shown). To evaluate the ability of gene expression under the control of the CMV promoter, we transfected the plasmid pValac::*anti-TNFα* into the HEK-293 cell line. The cell culture supernatant was collected 48 h post-transfection, and soluble scFv was probed with anti-HA primary antibody by western blot. A reactive band at the expected size of 31 kDa was detected, showing the production and secretion of the expected antibody fragment (Additional file 1: Figure S1B).

### Oral administration of the LL-FT strain ameliorates disease in DSS-induced colitis

**Fig. 1:**
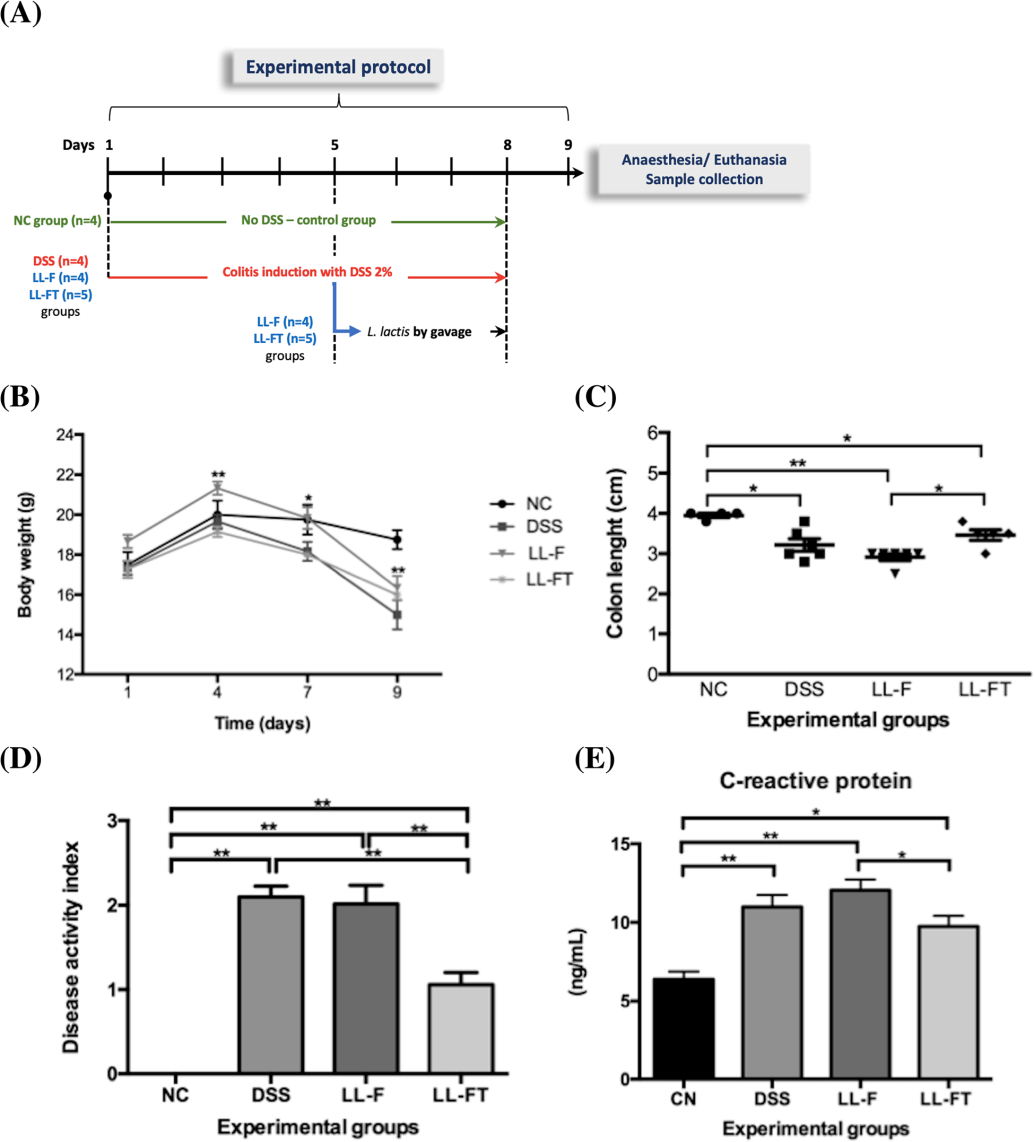
Effects of oral administration of LL-FT in DSS-induced colitis. **a** Experimental protocol is as follows: C57BL/6 mice received 2% DSS for 8 days (day 1 to day 8). Recombinant *L. lactis* was administered for 4 days (day 5 to day 8) to LL-F and LL-FT groups. Euthanasia and samples collection occurred on day 9 (arrow). **b** Body weight was measured in grams from day 1 to 9. **c** Colon length was measured in centimeters after euthanasia. **d** Disease activity index (DAI) was evaluated on day 9 before euthanasia and included three major clinical signs related to weight loss, diarrhea and rectal bleeding. **e** Blood serum of mice was collected after euthanasia and measured by ELISA. Experimental groups are as follows: NC, negative control group; DSS, DSS group; LL-F, *L. lactis* MG1363 FnBPA+ group and LL-FT, *L. lactis* MG1363 FnBPA+ (pValac::*anti-TNFα*) group. Data are expressed as the means ± SEM from an experiment using 4–5 animals per group. Statistical analysis was performed using the Mann-Whitney test for charts and two-way ANOVA for curves; * *p* < 0.05 and ** *p* < 0.01

The LL-F strain was transformed with the pValac::*anti-TNFα* plasmid by electroporation, and selected clones were checked by their restriction endonuclease digestion profile and PCR. The effects of LL-FT were evaluated in an animal model of acute colitis induced by DSS. An experimental protocol to mimic ulcerative colitis in humans was carried out. The mice received 2% DSS in drinking water for 4 days followed by a further 4 consecutive days of 2% DSS plus LL-F or LL-FT (Fig. 1a). As shown in Fig. 1b, body weight decreased with DSS ingestion, and there was no weight recovery in the mice that received LL-FT, despite clear clinical signs of improvement of inflammation, such as cessation of rectal bleeding and no signs of diarrhea. The colon length was examined after euthanasia. We found a shortening of the colons in the mice from the DSS group (average length of 3.2 ± 0.15 cm) in comparison with the group of mice that drank only saline (average length of 3.9 ± 0.05 cm). However, the group that received LL-FT showed a significant recovery of colon length (average length of 3.5 ± 0.13 cm) when compared with the LL-F group (average length of 2.9 ± 0.08 cm) (Fig. 1c). The effect of LL-FT on colitis in mice was evaluated using the disease activity index (DAI). This index reflects weight loss, diarrhea and rectal bleeding, parameters that had a milder occurrence in comparison with the LL-F group. This score was analyzed on day 9, before euthanasia. The mice treated with DSS alone without bacteria administration showed a score of 2.1 ± 0.13, and mice treated with LL-F showed a score of 2.0 ± 0.22. The mice that received LL-FT showed a significantly lower DAI of 1.1 ± 0.15 and thus a reduced inflammatory process compared to mice in the LL-F group (Fig. 1d). Moreover, CRP was measured (Fig. 1e). CRP levels increased among groups that received DSS, but the levels were significantly decreased in the LL-FT group, which reflects an anti-inflammatory effect.

### LL-FT prevents colonic mucosal injury

**Fig. 2:**
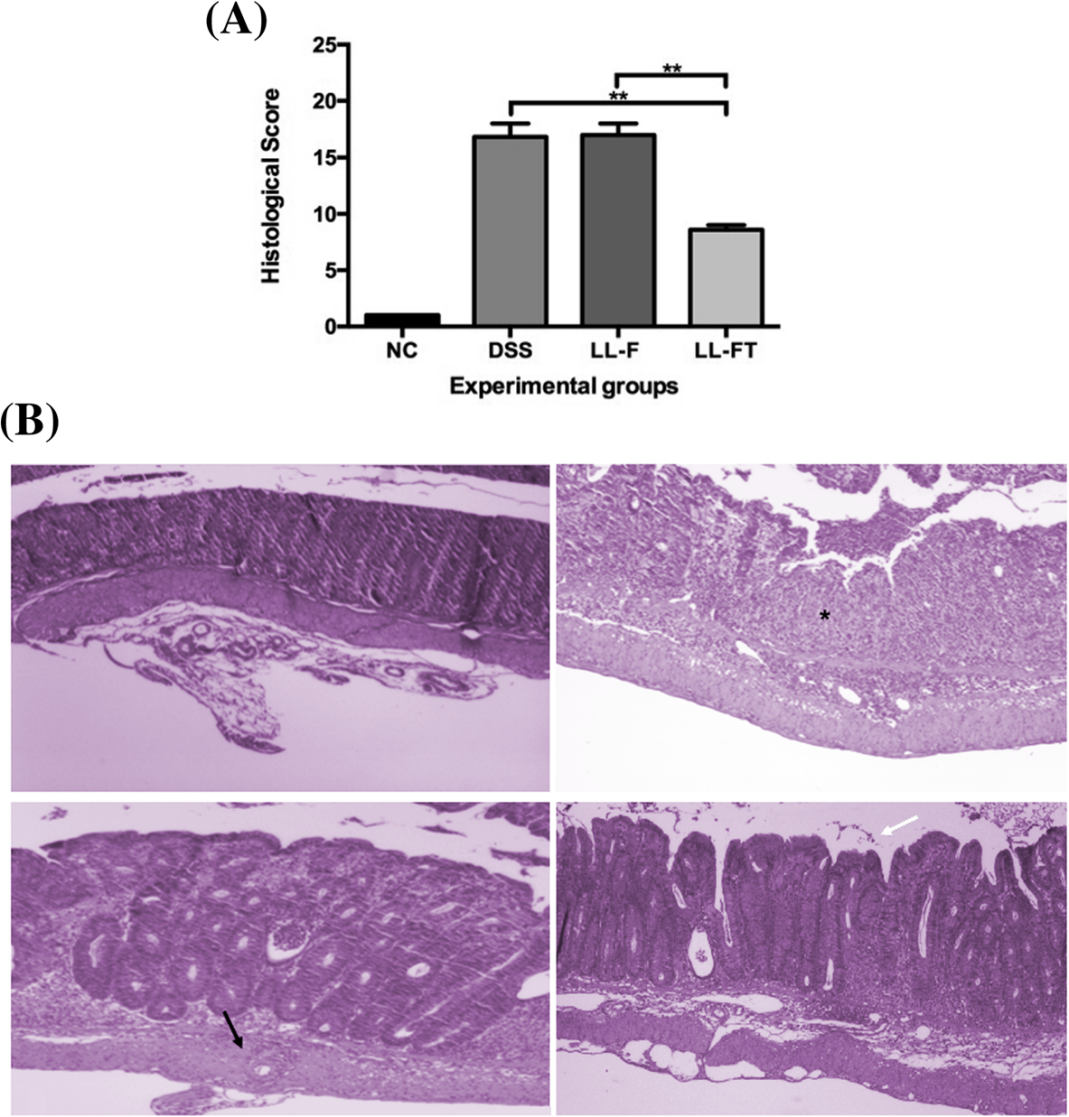
Histopathological score and histopathology of colonic tissue. **a** Histopathological score was determined from colon samples that were photographed in paraffin sections by H&E staining of a representative distal colon from each group. **b** Representative photos from distal colon tissue of a mouse from each experimental group are shown: *Star* ulceration; *black arrow* depletion of goblet cells; *white arrow* intestinal wall with intact mucosa and discrete inflammatory infiltrate. Experimental groups: NC, negative control group; DSS, DSS group; LL-F, *L. lactis* MG1363 FnBPA+ group and LL-FT, *L. lactis* MG1363 FnBPA+ (pValac::*anti-TNFα*) group. Data are expressed as the means ± SEM from an experiment using 4–5 animals per group and representative of three independent experiments. Statistical analysis was performed using the Mann-Whitney test; * *p* < 0.05 and ** *p* < 0.01

DSS induces extensive injury in the mouse colon [17]. Thus, histological analysis was performed for all animals to evaluate histological damage caused by DSS and *L. lactis* treatment. We developed a histological score considering several histopathological parameters (Additional file 1: Table S1). The histological scores for the LL-FT*-*treated animals were statistically significantly lower (*p* = 0.0022) compared with the scores of the DSS and LL-F groups (Fig. 2a). The colonic samples of negative control animals remained intact, with no change in the normal histological architecture in the mucosa (Fig. 2b NC). When comparing the mice in the LL-F group (Fig. 2b LL-F) to the mice in the DSS group (Fig. 2b DSS), the mucosal and submucosal inflammatory infiltrate ranged from moderate to severe. Furthermore, erosion with extensive ulceration, crypt abscesses, muscle herniation and depletion of goblet cells was observed. On the other hand, in the LL-FT group (Fig. 2b LL-FT), the mucosa, submucosa, muscular and serosal infiltrates were mild, with small or no erosion area and little gland inflammatory activity. In addition, mucosal ulceration or muscle thickening was not found, resembling the negative control of colitis. Hence, the presence of the *anti-TNFα* plasmid carried by *L. lactis* ameliorated the inflammatory symptoms, suggesting the participation of locally produced anti-TNFα.

### Modulation of inflammatory gene markers suggests disease reversal

**Fig. 3:**
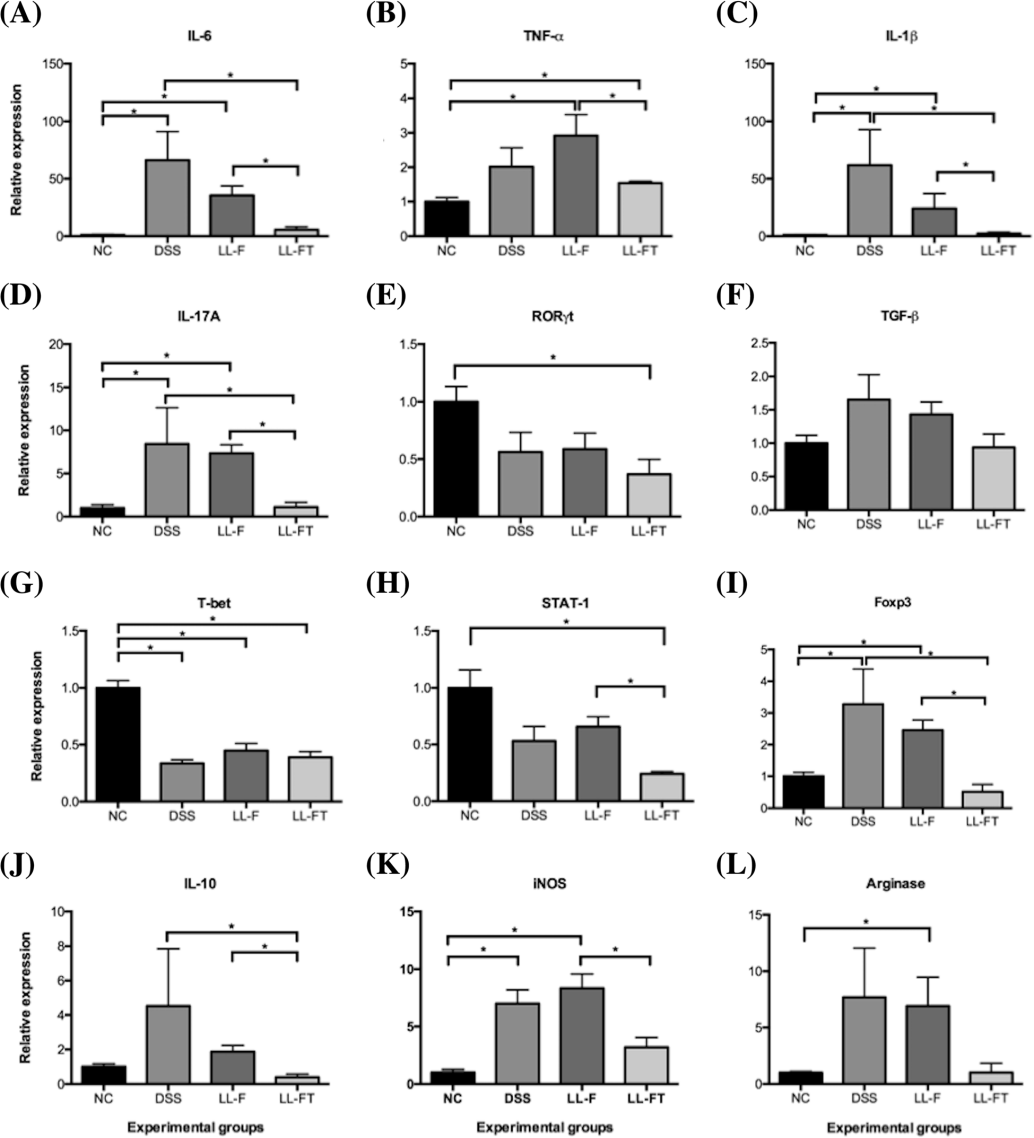
Effects of oral administration of LL-FT on mRNA expression levels in colonic tissue. Levels of mRNA were normalized to RPS9 mRNA. **a** IL-6, **b** TNFα, **c** IL-1β, **d** IL-17A, **e** RORγt, **f** TGF-β, **g** T-bet, **h** STAT-1, **i** Foxp3, **j** IL-10, **k** iNOS, and **l** Arginase. Experimental groups: NC, negative control group; DSS, DSS group; LL-F, *L. lactis* MG1363 FnBPA+ group and LL-FT, *L. lactis* MG1363 FnBPA+ (pValac::*anti-TNFα*) group. Data are expressed as the means ± SEM from an experiment using 4–5 animals per group. Statistical analysis was performed using the Mann-Whitney test; * *p* < 0.05 and ** *p* < 0.01

Cytokines and transcription factors involved in the mucosal immune response to colitis in mice were investigated by qPCR of the colonic total RNA. The mice treated with LL-FT were compared to control mice for the production of markers of T-cell and macrophage populations to investigate if anti-TNFα delivered by recombinant *L. lactis* could evoke specific sets of immune cell populations. As shown in Fig. 3, we found that the mRNA levels of the pro-inflammatory cytokines IL-6, TNFα and IL-1β were induced in mice that received DSS but that they significantly decreased in mice treated with LL-FT towards mRNA levels found in healthy animals (Fig. 3a, b, c). Similarly, IL-17A levels increased in DSS and LL-F groups and decreased to healthy levels after LL-FT treatment (Fig. 3d). RORγt mRNA levels decreased compared to those in untreated animals after colitis induction or *L. lactis* treatment. However, no statistical significance was observed except for the LL-FT group (Fig. 3e). Similarly, TGF-β mRNA levels did not change significantly by DSS or *L. lactis* treatment despite a small increase after DSS treatment (Fig. 3f). The relative expression of the Th1 marker T-bet decreased significantly in all groups that received DSS (DSS, LL-F and LL-FT group) compared with that of the NC group (Fig. 3g). Additionally, STAT1 mRNA levels were significantly lower in the LL-FT group in comparison to those in the NC and LL-F groups (Fig. 3h) suggesting a marked effect of LL-FT on the expression of STAT1 in the colon tissue. The Foxp3 expression increased in groups that received DSS and decreased significantly in mice treated with LL-FT (Fig. 3i), showing a profile similar to IL-17A. Likewise, the IL-10 anti-inflammatory cytokine transcripts were increased after DSS but decreased in the LL-FT group, showing significant differences (Fig. 3j). iNOS expression increased in groups that received DSS or DSS plus LL-F but decreased significantly in the LL-FT group (Fig. 3k). The arginase mRNA levels were similar to iNOS levels, but statistically significant differences only occurred between the NC group and the LL-F group, and the LL-FT group which received the recombinant *L. lactis* with pValac::*anti-TNFα* seemed to recover the NC group levels (Fig. 3l). We also tested MUC-3 levels, but they were not affected by DSS or *L. lactis* treatment (Additional file 1: Figure S2).

In addition to the mRNA transcripts, we evaluated if LL-FT could alter the serum levels of cytokines in mice. The cytokines were quantified at the end of the experiments on day 9. Levels of IL-6, TNF and IL-10 increased with DSS administration followed by a decrease when mice were treated with LL-FT, repeating the tendency of cytokine mRNA levels (Additional file 1: Figure S3). However, only TNF levels in the DSS group were significantly different compared to those in the LL-FT group. Thus, corroborating with a return to homeostasis after treatment.

Because IgA on the mucosal surfaces is considered the first line of defense controlling the pro-inflammatory processes and preserving the integrity of the epithelial barrier [18, 19], we measured IgA levels in the fecal extracts of mice to verify the integrity of the mucosal tissue following treatment. We observed that mice receiving DSS presented higher IgA levels than the negative control group and that treatment with LL-F did not rescue untreated levels. However, no significance was observed at *p* < 0.05 (Additional file 1: Figure S4). On the other hand, LL-FT treatment showed a tendency to return IgA levels to untreated levels.

## Discussion

In this work, we showed the construction of an anti-TNFα scFv eukaryotic expression vector to be delivered by oral administration to the gut of animals. This vector is based on a previously described vector (pValac) that allows the expression of a transgene in gut epithelial cells [16]. We showed that this vector induces the synthesis of a 31 kDa scFv in transfected HEK-293 cells, suggesting its competence to induce synthesis in the gut cells of animals. Therefore, we expected to delivery an anti-TNFα in the gut of mice via a genetically modified microorganism, *L. lactis*. We tested this hypothesis by treating mice suffering from ulcerative colitis induced by DSS, an experimental disease model known to be ameliorated by anti-TNF therapy. Furthermore, we tested the use of LAB as a delivery system for antibody fragments as an alternative strategy for the treatment of IBD.

Wild-type *L. lactis* are able to be internalized by eukaryotic cells and to deliver DNA efficiently, as shown previously both in vitro [20] and in vivo [21]. However, a recombinant *L. lactis* expressing a fibronectin-binding protein A (FnBPA) of *Staphylococcus aureus* has an improved ability to deliver DNA since FnBPA is an invasin that mediates invasivity in nonphagocytic host cells [22], facilitating intracellular spreading [15]. Therefore, for this study, we used a modified bacteria transformed with the plasmid pValac::*anti-TNFα* to adheres to eukaryotic cells of the intestinal mucosa [23]. Thus, LL-F transformed with the pValac::*anti-TNFα* (LL-FT) plasmid was administered by gavage to a DSS-induced colitis in mice.

DSS is a high molecular weight and irritating chemical known to induce colitis. At a concentration of 2%, it causes severe inflammation in the mucosa of the intestinal colon [24]. After receiving DSS, the mice presented distinguished weight loss, diarrhea and rectal bleeding. C57BL/6 mice were used in this work because this strain is highly susceptible to DSS colitis and because these mice do not evolve spontaneously to healing but rather develop a chronic state [25, 26]. In our model, mice exhibited the same disease characteristics, and after the treatment with LL-FT, we showed improvement in a variety of macroscopic parameters. The shortening of the colon length reflects the inflammatory process, and the treatment of mice with LL-FT improved the colon length compared to that of the LL-F group. Moreover, the same group LL-FT showed no significant difference compared to the untreated group. However, the macroscopic score (DAI), which reflects multiple macroscopic pathological parameters, showed that the mice that received the *anti-TNFa* transgene had a lower DAI than other control groups, suggesting a reversal of the disease.

The histopathological score as computed here gives an assessment of the microscopic aspect of the pathological process. The colon of the untreated animals appeared healthy, showing an integral histological architecture with visible microvillosities. This healthy aspect was lost after DSS treatment. Moreover, our experiments of inducing acute colitis by DSS was reproduced since, histologically, we found lesions with inflammatory infiltrate, focal crypt lesions and goblet cell loss [27]. The damage induced by DSS was not reduced after treatment with LL-F; however, a statistically significant benefit to tissue integrity was observed in mice receiving LL-FT. Therefore, the beneficial effect of LL-FT treatment most be the consequence of the anti-TNFα transgene expression after *L. lactis* treatment. The findings in the histopathology of the colon agreed with the macroscopic parameters and histopathological scores observed by other authors [26], and *L. lactis* harboring anti- TNFα treatment seemed to ameliorate colon tissue integrity. It is noteworthy that even though we are describing the results of a single experiment, these effects were reproducible by at least three independent experiments (data not shown).

To follow the inflammatory response of our model, we measured CRP, an acute-phase protein produced by the liver in acute inflammatory conditions and is a useful marker of IBD [28]. Although CRP is a nonspecific mucosal inflammatory marker, our data indicate that lower levels of this protein were associated with a lower active disease index in mice and could be used as a functional biomarker for the evaluation of intestinal inflammation. It is noteworthy that FnBPA acts as an inflammatory marker per se [29] that may counteract in part the beneficial effect of LL-F or LL-FT. Therefore, the administration of *L. lactis* delivering anti-TNFα after the fourth day of DSS treatment improved the overall aspect of disease, suggesting a reversal of the DSS-induced process.

DSS-induced colitis disrupts the epithelial barriers, allowing intestinal bacteria to invade the damaged mucosa and inducing excessive production of pro-inflammatory cytokines [30] that could be reduced with anti-TNF therapy [6]. In our model system, the mRNA levels of pro-inflammatory cytokines such as IL-6, TNFα and IL-1β increased after disease induction, as expected [31, 32] and decreased to levels similar to those of healthy controls after LL-FT treatment. IL-17A levels respond similarly, showing a clear upregulation after disease induction, supporting the Th17 axis in the experimental murine colitis model [32–35]. Coherently, T-bet, a hallmark of the Th1 response, was not affected by recombinant bacteria, corroborating an immune response associated with the Th17 phenotype. Regulatory T cells (Treg) are able to suppress abnormal immune responses, and they are involved in homeostasis of the intestinal mucosa [36]. These cells, marked by the expression of Foxp3, produce IL-10 and TGF-β and inhibit the effector function of T cells [3]. Treg markers are increased in the gut of CD and UC patients [37]. Our data suggest that mice treated with LL-FT recover to healthy levels of Foxp3 and IL-10, suggesting a reduction in Tregs, which possibly reflects the improvement in the disease index after treatment with anti-TNFα and a return to homeostasis.

Gobert et al. [38] showed an amelioration of colitis symptoms induced by DSS in iNOS-deficient mice, and our data showed a significant decrease in mRNA iNOS when mice were treated with LL-FT, suggesting that treatment exerted a beneficial effect. Arginase, an M2 macrophage-associated gene, also showed an increase when mice were exposed to DSS followed by a decrease with recombinant *L. lactis*. The association of increased arginase and increased IL-10 suggests that there is an accumulation of M2 macrophages in the colonic tissue of DSS-induced colitis as described by Lin et al. [39], and a lowering of these markers after LL-FT treatment leads to the resolution of the inflammatory process.

The homeostasis of the gastrointestinal tract is achieved by immune mechanisms such as secretion of mucus and IgA, which protects the intestinal epithelium against commensal and pathogenic microorganisms. MUC-3 codes for a structural protein are involved in the healthy epithelium. Some authors showed that MUC-3 is upregulated during the recovery of a damaged bowel after acute colitis [40]. In our model, MUC-3 seems to be downregulated, even though there was no statistical significance. IgA immunoglobulin is the most abundant isotype produced by the mucosa and constitutes an effective marker of inflammation within the microbiota, controlling and modulating it [41, 42]. In our model, the administration of LL-FT did not significantly increase IgA levels in the intestine. Surprisingly, those animals treated with DSS and received saline or LL-F without plasmid had high levels of IgA in comparison with the negative control group and treatment group. Souza et al. [13] also found no increase in IgA levels in mice that received doses of LL-F. On the other hand, a study showed that the use of this same strain carrying pValac::*il-10* was associated with high IgA levels when compared to those in the DSS group [14]. Despite the lack of statistical significance, our results suggest that the induction of disease shows a tendency to increase intestinal IgA levels compared to levels in healthy and anti-TNFα-treated groups. This increase may reflect disease activity directly or indirectly since IgA may be lacking from plasma due to colon tissue damage associated with DSS treatment.

Several cytokines are known to be involved in IBD [43, 44], therefore their serum concentrations were evaluated, and the results corroborated with local mRNA levels, validating the data achieved via qPCR. Even though we see scarce significant differences, the excessive secretion of pro-inflammatory cytokines is related to intestinal inflammation [3, 26, 37, 43], and this biological phenomenon seemed to be reproduced in our model system. The significative reduction of the systemic levels of TNFα could be reflecting a neutralization of this cytokine, hitherto a key player of the inflammatory process in human colitis. Infliximab and adalimumab [45], both anti-TNFα monoclonal antibodies, are currently used for treating Crohn’s disease. Moreover, it lessens DSS induced colitis in mice [46, 47]. However, recent data suggests that neither infliximab binds TNFα [48], nor TNFα is necessary for colitis in TNFα^−/−^ mice [49] contradicting current admitted mechanism for infliximab action on mouse model based on TNFα neutralization [6, 50, 51]. Despite of the mechanism, infliximab ameliorates DSS induced colitis that is corroborated by our results. It is possible that other mechanism, such as an apoptosis based one may underlie colitis mouse models response to anti-TNFα therapy [52–54], not TNFα neutralization. Consistently, the observed reduction of systemic TNFα observed in our experiment correlated to a decline in its mRNA levels, thus, TNFα seemed to decline due to a reduction of synthesis rather than from neutralization.

Anti-TNFα therapies have become popular for treating UC and CD in humans, and mucosal delivery of biopharmaceuticals may improve the outpatient’s quality of life. However, the oral delivery of antibodies is hindered by the harsh conditions of the digestive tract. Thus, strategies based on mucosal delivery by microorganisms may overcome this obstacle by producing the antibody directly in the gut. We show here that genetically engineered *L. lactis* can be used to deliver an scFv anti-TNFα to the mammalian intestine. Vandenbroucke et al. [6] had previously reported the use of anti-TNF nanobodies secreting *L. lactis* in DSS-induced colitis in the IL-10^−/−^ mouse, where they found the resolution of the inflammatory process. Their system was based on a single-domain camelid antibody fragment constitutively secreted from the bacteria. The expression system used here is based on the heterologous production of an scFv vector directly in the epithelium of the intestine in a eukaryotic expression system [16], restricting anti-TNFα to the gut milieu focusing the therapeutic intervention.

The mucosal delivery may help targeting immune modulation towards the gut, but some restriction could be pointed. The chemical instability of scFv could be partially overwhelmed by its in loco production drove by a mammalian expression system, but the amount of bioavailable pharmaceutical is still unpredictable. Therefore, finding an effective and reproducible dose of bacteria may be a challenging issue. Moreover, the use of recombinant proteins associated with symbiotic microbiota is only starting to be investigated [55], and variable results may be observed with non-conventional delivery, as observed with a bacterial membrane associated anti-TNF delivery [56].

In the present report, the scFv anti-TNFα was engineered inspired on the well-studied agent infliximab [57] and was delivered directly by cells in the gut. Hence, the delivery system proposed here may represent a more reliable model system for simulated anti-TNF treatment for UC in human subjects. Because this system uses a noninvasive route to carry the biopharmaceutical to the site of inflammation, it may represent an alternative for oral antibody therapies.

## Conclusions

The use of LAB for delivering biopharmaceuticals may represent an alternative route for immunotherapy. The results reported here suggests that oral administration of *Lactococcus lactis* carrying the eukaryotic expression vector coding for an anti-TNFα induces a reduction of colitis associated inflammatory and histopathological markers suggesting an amelioration in disease. Novel therapeutic approach based on delivering recombinant antibodies may soon substitute systemic immunotherapy for gut-associated diseases.

## Methods

### Bacterial strains, media and growth conditions

*Escherichia coli* XL1-Blue and *E. coli* TG1 were grown in Luria-Bertani (LB) medium with tetracycline (Tet; Sigma-Aldrich, St. Louis, MO, USA) at 30 μg/mL (only for XL1-Blue) at 37 °C and 250 rpm overnight. *L. lactis* MG1363 FnBPA+ were grown in M17 medium (Difco, Detroit, MI, USA) supplemented with 0.5% glucose, and, when necessary, chloramphenicol (Cm; Sigma-Aldrich, St. Louis, MO, USA) at 10 μg/mL and erythromycin (Ery; Sigma-Aldrich, St. Louis, MO, USA) at 5 μg/mL, at 30 °C without agitation for 18 h. The plasmids and bacterial strains used are described in Table 1 [15, 16].

**Table 1:** Bacterial strains and plasmids used in this study

Plasmids	Characteristics	Reference
pValac	Eukaryotic expression vector (pCMV/Cm^r^/RepA/RepC)	[16]
pValac::*anti-TNFα*	pValac containing *anti-TNFα* scFv ORF	This study.
Bacterial strain and plasmids	Characteristics	Reference
*Escherichia coli* XL1-Blue	*E. coli*; F′[*Tn10 proAB*^*+*^ *lacI*^*q*^ *Δ (lacZ)M15*] *hsdR17(r*_*K*_^*−*^ *m*_*K*_^*+*^)/ Tet^r^	Stratagene® (Catalog n^o^ #200249)
*Escherichia coli* TG1	*E. coli,* K-12-derived strain; F′ [*traD36 proAB*^+^ *lacI*^*q*^ *lacZΔM15*] *supE thi-1* Δ (*lac-proAB*) Δ (*mcrB-hsdSM*)*5*, (*r*_*K*_^*−*^*m*_*K*_^*−*^)	Lucigen, Middleton, MI, USA (Catalog n^o^ 60502–1)
*Lactococcus lactis* MG1363 FnBPA+ (LL-F)	*L. lactis* MG1363 strain expressing FnBPA of *S. aureus* (Ery^r^)	[15], obtained from Laboratory of Cellular and Molecular Genetics (LGCM), Federal University of Minas Gerais (UFMG), Brazil,
*Lactococcus lactis* MG1363 FnBPA+ (pValac::*anti-TNFα*) (LL-FT)	*L. lactis* MG1363 FnBPA+ strain carrying the pValac::*anti-TNFα* plasmid (Cm^r^/ Ery^r^)	This study.

Cm^r^ chloramphenicol resistance; Tet^r^ tetracycline resistance; Ery^r^ erythromicin resistance

### Construction of pValac::*anti-TNFα* and development of LL-FT

An anti-TNFα scFv expression vector was constructed based on infliximab variable chain sequences (GenBank accession numbers: 471270577 and 471,270,576). A synthetic scFv was designed based on mouse codon usage and cloned in the pValac shuttle vector. The synthetic gene fragment was cloned into the pValac::*gfp* vector digested with *Eco*R I (Invitrogen, Carlsbad, CA, USA) and *Nhe* I (Invitrogen, Carlsbad, CA, USA), yielding pValac::*anti-TNFα*. An HA tag was included in the carboxy terminus of the scFv. This vector was used to transform *E. coli* TG1 as described by Sambrook and Russel [58]. Cloning was checked via the restriction endonuclease digestion profile, polymerase chain reactions (PCR) and sequencing [59], confirming the integrity of sequences. LL-F was transformed with pValac::*anti-TNFα* using electroporation as previously described [60], resulting in LL-FT. The presence of plasmid was confirmed by PCR and enzymatic digestion.

### Transfection assays of mammalian HEK-293 cells with pValac::*anti-TNFα*

The pValac::*anti-TNFα* plasmid was tested for anti-TNFα protein expression by human embryonic kidney (HEK-293) cell line transfection. HEK-293 cells (ATCC number: CRL-1573™) obtained from Rio de Janeiro Cell Bank (Rio de Janeiro, Brazil), were cultured in Dulbecco’s modified Eagle’s medium (DMEM) (Gibco®, Glasgow, UK) supplemented with 10% fetal bovine serum (FBS) (Gibco®, Glasgow, UK) and Antibiotic-Antimycotic (100X) (Gibco®, Glasgow, UK) at 37 °C in 5% CO_2_. Lipofectamine™ LTX reagent (Invitrogen, Carlsbad, CA, USA) was used for the transfection assay with 2 μg of pValac::*anti-TNFα* in wells containing 70–90% confluent cells according to the manufacturer’s recommendations. After 48 h, samples were centrifuged at 10,000×g for 5 min, and the supernatant was stored at − 20 °C until anti-TNFα detection by western blot.

### SDS-PAGE and Western blot analysis

Proteins from the supernatant of HEK-293 transfection were resolved by 12% SDS-PAGE using the Bio-Rad system (Bio-Rad, Hercules, CA, USA) for electrophoresis. Proteins were transferred to a nitrocellulose membrane (GE Healthcare, Uppsala, Sweden) that was blocked with PBST-milk (PBS buffer added 5% skim milk and 0.1% Tween 20) and then incubated with anti-HA probe (1:1000; Sigma-Aldrich, St. Louis, MO, USA). After PBST washing, the membrane was incubated with alkaline phosphatase-conjugated anti-rabbit IgG antibody (1:5000; Southern Biotechnology, Birmingham, AL, USA). After washing with PBST and APB, enzymatic activity was performed using a BCIP/ NBT chromogenic substrate (Invitrogen, Carlsbad, CA, USA).

### Mice

Conventional female C57BL/6 mice [61] (10 week) were purchased from the CEMIB (Centro Multidisciplinar para Investigação Biológica) of Universidade Estadual de Campinas (Unicamp – Campinas, Brazil). Animals belonging to the same experimental group were housed in a single cage in a controlled temperature (25 °C) room with a 12:12-h light/dark cycle and ad libitum access to food and water. Sample number estimation in each experimental group and all animal procedures were performed following the rules of the Ethical Principles in Animal Experimentation adopted by the Ethics Committee on Animal Experimentation (CEUA/ICB-UnB/Brazil) and approved by CEUA (51,069/2015).

### DSS-induced colitis and treatment with *L. lactis* MG1363 FnBPA+ (pValac::*anti-TNFα*)

Acute colitis was induced by adding 2% (w/v) DSS (MW 40–50 kDa; USB Affymetrix, Santa Clara, CA, USA) to the drinking water from day 1 to day 8 [24]. Experiments were carried out with 4 to 5 mice per group. The mice were divided into the following groups: i) a healthy negative control group (NC) in which the mice were gavaged with 100 μL of saline (0.9% NaCl) and allowed to ingest pure filtered water throughout the experiment, ii) a positive control group of colitis (DSS) in which the mice were gavaged with 100 μL of saline, iii) the LL-F group in which the mice received 100 μL of the corresponding bacterial strain as suspension without plasmid, and iv) the LL-FT group in which the mice received 100 μL of the corresponding bacterial strain as suspension. The DSS, LL-F and LL-FT groups ingested filtered water with 2% DSS added throughout the experiment. The recombinant strains were administered once daily by gavage from day 5 to day 8. Each dose corresponded to 100 μL of LL-FT suspension and contained 2.0–2.5 × 10^9^ colony forming units (CFU). Animals were euthanized on day 9 in a 5% carbon dioxide chamber with cervical dislocation to collect blood samples from the retro-orbital venous plexus; the colonic tissue was quickly removed and washed of feces. Animals that died during the experiment were not included in the analysis. The mean of the water-DSS intake per group was monitored, and each animal consumed 3–5 mL of water daily.

### Disease activity index (DAI)

On day 9 (day of euthanasia), DSS-induced colitis was determined using the disease activity index (DAI) as described by Cooper et al. [62]. The DAI consisted of the combined scores for weight loss, stool consistency and rectal bleeding divided by 3. The features that were graded included the following: body weight loss (0, none; 1, 1–5% loss; 2, 5–10% loss; 3, 10–20% loss; and 4, > 20% loss), stool consistency (0, normal; 2, loose stools and 4, diarrhea) and rectal bleeding (0, absent; 2, moderate and 4, severe). Loss of body weight was defined by the difference between the initial and final weight. Stool consistency and rectal bleeding were confirmed by examination of the sectioned colon upon euthanasia.

### Quantification of C-reactive protein in blood serum

C-Reactive Protein (CRP) was quantified in the blood serum of animals with a Mouse CRP ELISA Kit (Sigma-Aldrich, St. Louis, MO, USA) according to the manufacturer’s instruction. After euthanasia, collected blood samples were kept at room temperature until coagulation and then centrifuged at 5000×g for 5 min. The serum samples (supernatant) were then transferred to new tubes and stored at − 20 °C. For ELISA, a dilution of 1:20,000 from each serum sample was used. The reading was performed at 450 nm on a VersaMax™ ELISA Microplate Reader (Molecular Devices, San Jose, CA, USA).

### Histopathological score of colitis

On the day of euthanasia, the colon was removed, and its length was measured. Distal colon samples were sectioned into two fragments to be used for histological study and biochemical determinations. Colonic tissue was fixed in 10% formaldehyde, and H&E staining was performed. The histological analyses were carried out in a blind design and were based on the morphological findings regarding the presence of inflammatory infiltrate reaching the mucosa, submucosa, muscular and serosal layers; inflammatory activities in glands; abscesses of crypts; erosion or ulceration of the mucosa; thickening of the muscular layer; and depletion of goblet cells and herniation of the muscular layer. These findings were classified as mild, moderate or severe. Thus, the following scores were assigned: 0, mucosa with normal structures, without any alteration or with slight inflammatory infiltrate in the mucosa or submucosa; 1, the presence of mild-to-moderate inflammatory infiltrate with inflammatory activity in glands or abscesses of crypts with erosion but without ulceration; 2, the presence of all the previous findings associated with greater ulcerations in the mucosa but one or two ulcers; and 3, the presence of ulcerations compromising large areas of the mucosa. The score of each animal could range from 0 to 39.

### RNA isolation and qPCR analysis

For gene expression analysis of the colonic samples by quantitative PCR (qPCR), the tissue was stored in RNAlater (Qiagen, Valencia, CA, USA) until total RNA extraction using a RNeasy Protect Mini Kit (Qiagen, Valencia, CA, USA) and TissueLyser LT (Qiagen, Valencia, CA, USA) to disrupt the samples. The samples were quantified using a NanoDrop™ One^C^ Microvolume UV-Vis Spectrophotometer (Thermo Scientific™, Waltham, MA, USA). For reverse transcription, RT^2^ First Strand Kit (Qiagen, Valencia, CA, USA) was used. For elimination of genomic DNA during RNA purification, RNase-Free DNase Set (Qiagen, Valencia, CA, USA) was used. Amplification and detection were performed on optical 96-well plates (Applied Biosystem, Foster City, CA, USA) with the 7500 Fast Real Time PCR System (Applied Biosystem, Foster City, CA, USA) using a Fast SyBR Green Master Mix Kit (Applied Biosystem, Foster City, CA, USA). Levels of mRNA expression were normalized to ribosomal protein S9 (RPS9) mRNA, and RNA relative quantification was calculated using the method 2^-ΔCt^ [63]. The oligonucleotide primers are described in Additional file 1: Table S2.

### Analysis of blood serum cytokines by flow cytometry

The sera of mice were collected to measure the levels of interleukin-6 (IL-6), TNF and IL-10 using a Cytometric Bead Array (CBA) Mouse Inflammation Kit (BD Biosciences, San Jose, CA, USA) as recommended by the manufacturer. Samples were acquired in an Accuri™ C6 flow cytometer (BD Biosciences, San Jose, CA, USA) and analyzed using FCAP Array™ Software version 3.0 (BD Biosciences, San Jose, CA, USA). At least 2100 events were acquired for each sample.

### Measurement of fecal IgA

Levels of IgA were determined in fecal extract samples using a Mouse IgA ELISA Kit (Sigma-Aldrich, St. Louis, MO, USA) following the manufacturer’s protocol. Before euthanasia, the mice were placed in clean cages and kept for 30 min. A pool of feces per group was collected, weighed, transferred to a Falcon tube containing 5 mL of PBS added to 100 mM PMSF 0.2% and kept on ice for 15 min. The extracts were homogenized via inversion of the tube and kept on ice for 15 min. Then, the tubes were centrifuged for 30 min at 3000 rpm at 4 °C. The supernatants were saved and stored at − 80 °C. For ELISA, dilutions of 1:10 and 1:100 from each pool were used. The reading was performed at 450 nm on a VersaMax™ ELISA Microplate Reader (Molecular Devices, San Jose, CA, USA).

### Statistical analysis

The results of experiments are expressed as the means ± SEM. Statistical differences were determined by two-way ANOVA with a Bonferroni post hoc test for curves and by the Mann-Whitney test for charts. All statistical analyses were performed with Graph Pad Prism version 6.0 for Mac OS X (La Jolla, CA, USA). Statistical significance was considered at *p* < 0.05.

## Additional files

Additional file 1: **Figure S1.** Structure of the eukaryotic expression vector pValac::*anti-TNFα* and scFv protein expression by HEK-293 on transfection assays. **Figure S2.** MUC-3 mRNA levels in colonic tissue. **Figure S3.** Effect of the treatment of colitis with LL-FT on systemic cytokines production. **Figure S4.** Changes in fecal IgA after oral administration of LL-FT. **Table S1.** Primer sequences used in qPCR assay. **Table S2.** Histological Score. (DOCX 684 kb)

## Abbreviations

CBA: Cytometric bead array
CD: Crohn’s disease
CFU: Colony forming units
CRP: C-reactive protein
DAI: Disease activity index
DSS: Dextran sulfate sodium
FBS: Fetal bovine serum
FnBPA: Fibronectin-binding protein A
GRAS: Generally regarded as safe
IBD: Inflammatory bowel disease
LAB: Lactic acid bacteria
LL-F: *Lactococcus lactis* MG1363 FnBPA+
LL-FT: *Lactococcus lactis* MG1363 FnBPA+ (pValac::*anti-TNFα*)
ORF: Open read frame
PCR: Polymerase chain reactions
qPCR: Quantitative PCR
scFv: Single-chain fragment variable
TNFα: Tumor necrosis factor-α
UC: Ulcerative colitis
