# Supplementary material for: Mucosal delivery of Lactococcus lactis carrying an anti-TNF scFv expression vector ameliorates experimental colitis in mice
Source: BMC Biotechnol. 2019 Jun 25;19:38. doi: 10.1186/s12896-019-0518-6 (PMC6593574; doi:10.1186/s12896-019-0518-6)
Supplement: Supplementary file 1 — Figure S1. Structure of the eukaryotic expression vector pValac::anti-TNFα and scFv protein expression by HEK-293 on transfection assays. Figure S2. MUC-3 mRNA levels in colonic tissue. Figure S3. Effect of the treatment of colitis with LL-FT on systemic cytokines production. Figure S4. Changes in fecal IgA after oral administration of LL-FT. Table S1. Primer sequences used in qPCR assay. Table S2. Histological Score. (DOCX 684 kb) [file 12896_2019_518_MOESM1_ESM.docx]

Additional File 1

**Mucosal delivery of an anti-TNF scFv expression vector carried by** *Lactococcus lactis* **ameliorates experimental colitis in mice**

Maria José Chiabai^1^,

Juliana Franco Almeida^2^,

Mariana Gabriela Dantas de Azevedo^1^,

Suelen Soares Fernandes^1^,

Vanessa Bastos Pereira^3^

Raffael Júnior Araújo de Castro^4^,

Márcio Sousa Jerônimo^4^,

Isabel Garcia Sousa^1^,

Leonora Maciel de Souza Vianna^5^,

Anderson Miyoshi^3^,

Anamelia Lorenzetti Bocca^4^,

Andrea Queiroz Maranhão^1^,

Marcelo de Macedo Brigido^1^

**Correspondence:** Marcelo Macedo Brigido: [brigido@unb.br](mailto:brigido@unb.br)

# Additional Figures

**(A) (B)**

**
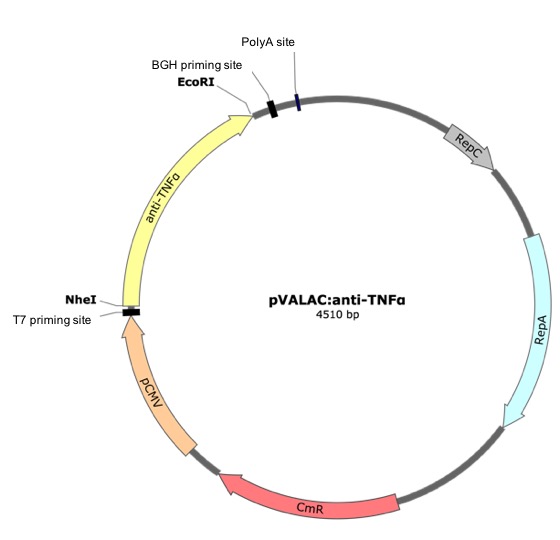

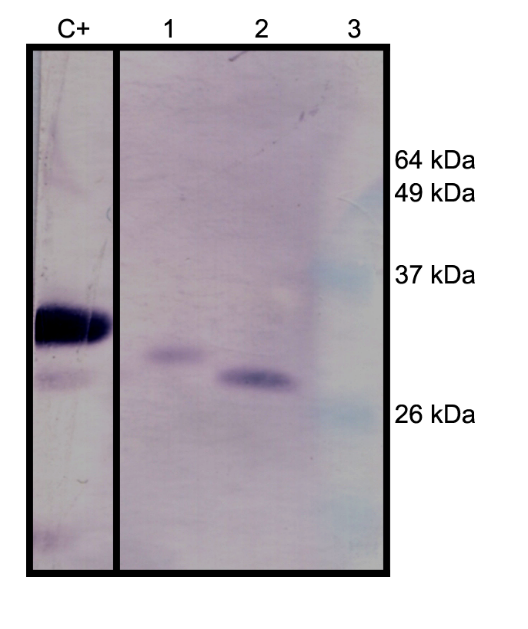
**

**Figure S1 Structure of the eukaryotic expression vector pValac::*anti-TNFα* and scFv protein expression by HEK-293 on transfection assays.** **(A)** Schematic representation of pValac::*anti-TNFa* plasmid harboring a cytomegalovirus promoter (pCMV), *anti-TNFα* ORF of *Mus musculus,* a polyadenylation signal of bovine growth hormone (BGH polyA), which are essential for expression of gene by eukaryotic host cells; *E. coli* (RepC) and *L. lactis* (RepA) origins of the replication to the prokaryotic region and chloramphenicol resistance gene (Cm) for bacterial selection. **(B)** Western blot shows the presence of scFv anti-TNFα (31 kDa) produced in the posttransfected supernatant of HEK-293 cell cultures. C+, scFv positive control; 1, supernatant of HEK-293 producing anti-TNFα; 2, scFv anti-IL1β positive control (30 kDa); and 3, BenchMark^TM^ Pre-Stained Protein Ladder (Invitrogen, Carlsbad, CA, USA).


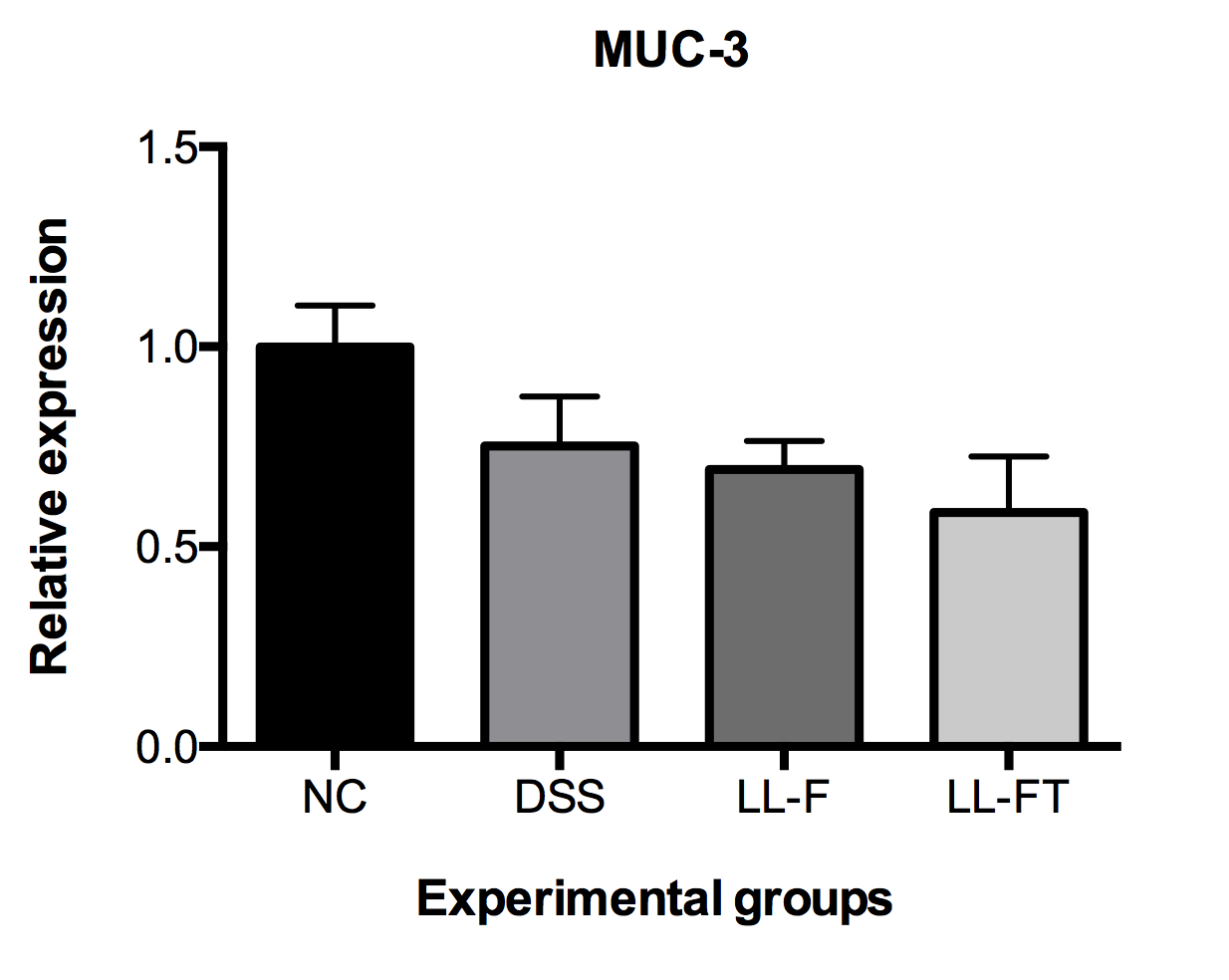


**Figure S2 MUC-3 mRNA levels in colonic tissue.** Levels of mRNA were normalized to RPS9 mRNA. Experimental groups: NC, negative control group; DSS, DSS group; LL-F, *L. lactis* MG1363 FnBPA+ group and LL-FT, *L. lactis* MG1363 FnBPA+ (pValac::*anti-TNFα*) group. Data are expressed as the means ± SEM from an experiment using 4-5 animals per group. Statistical analysis was performed using the Mann-Whitney test.

1. **(B) (C)**


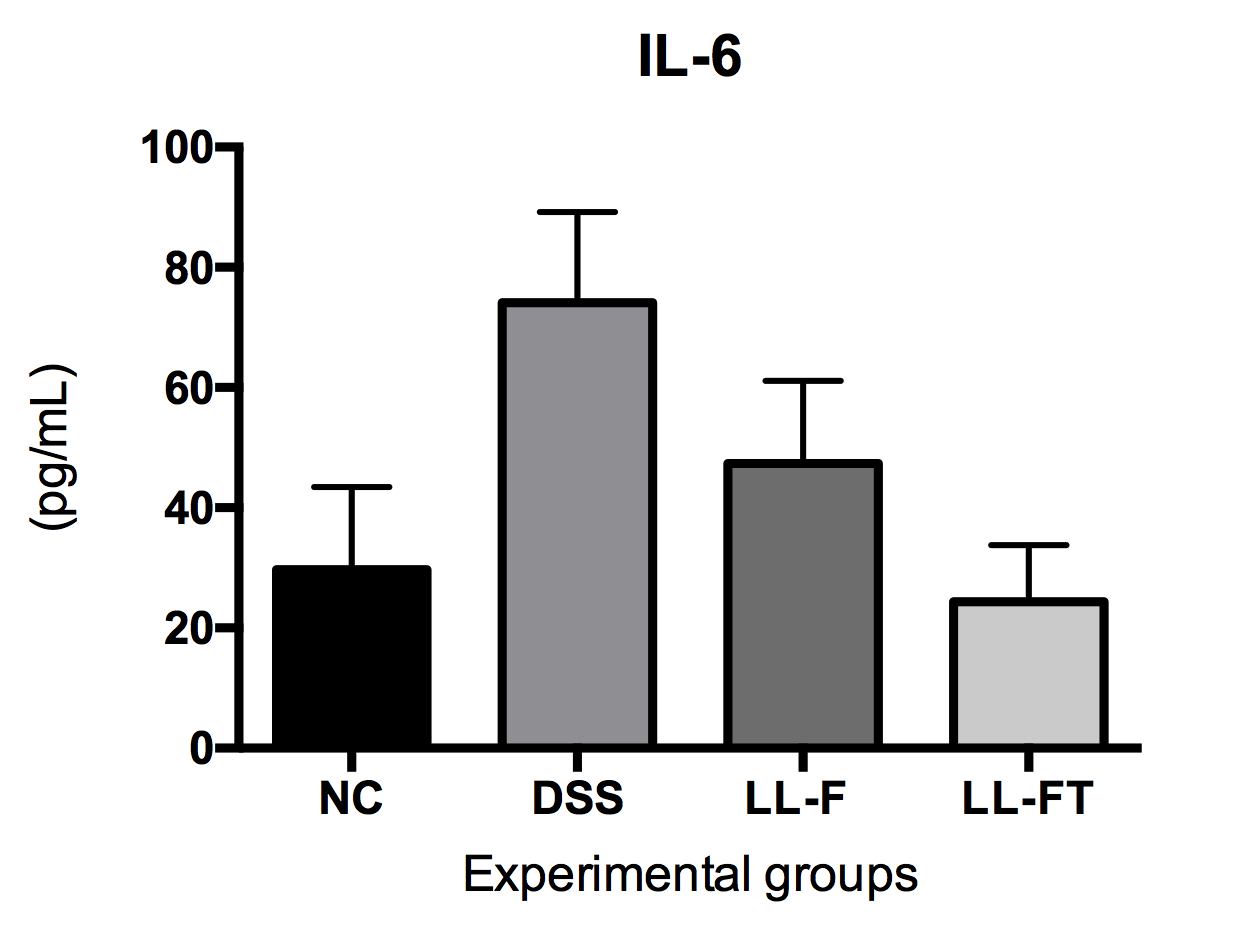

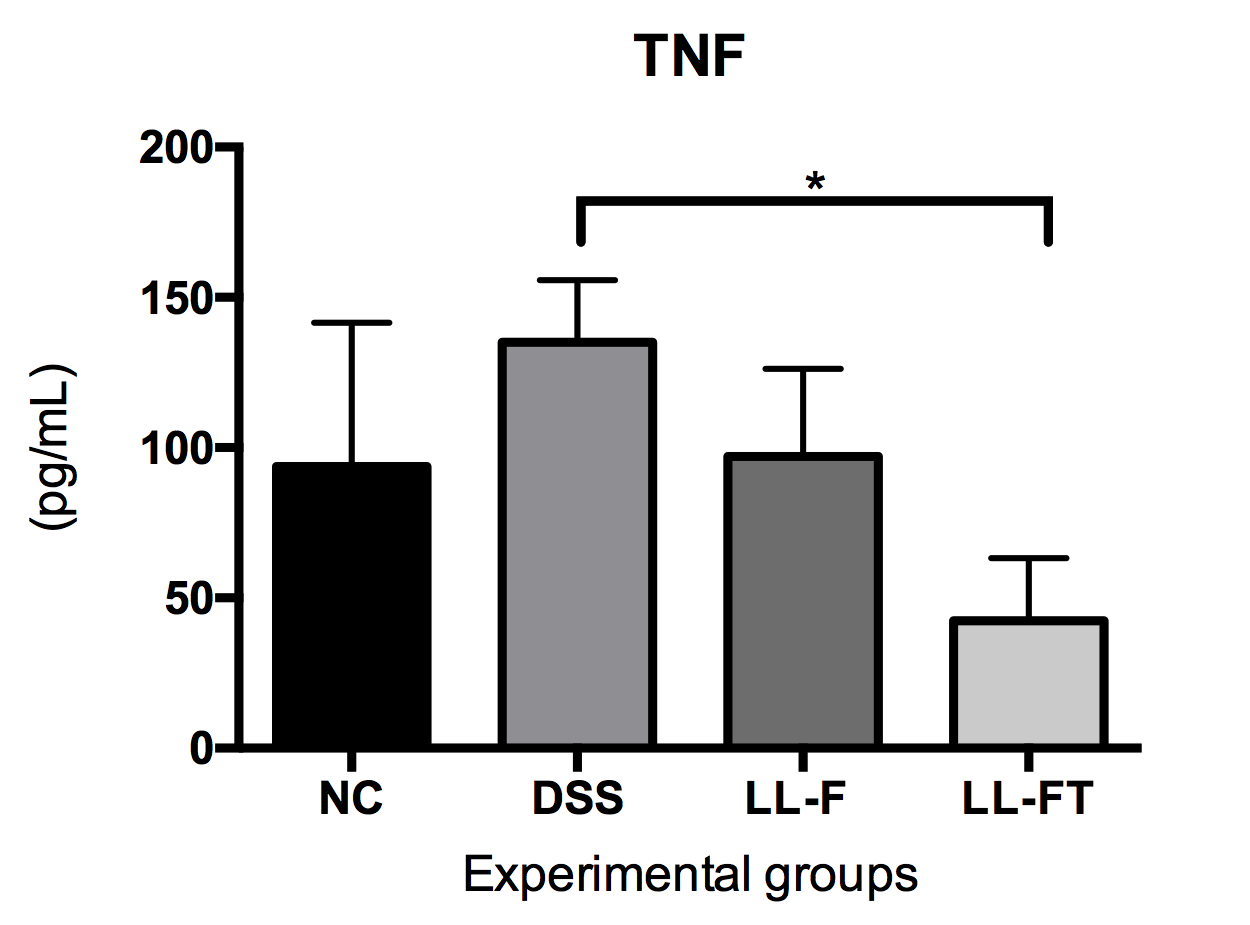

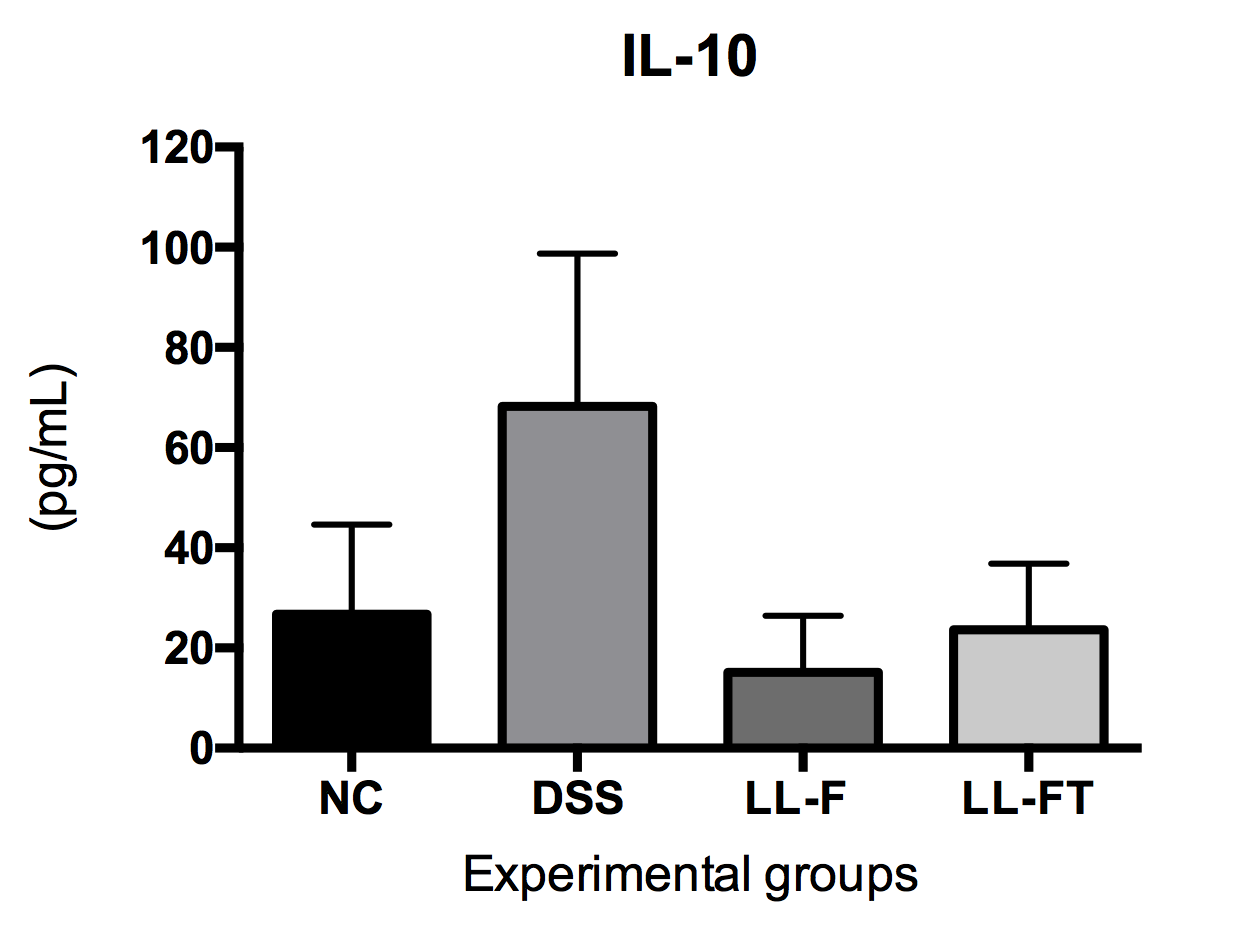


**Figure S3 Effect of the treatment of colitis with LL-FT on systemic cytokines production.** Systemic **(A)** IL-6, **(B)** TNF, and **(C**) IL-10 production levels in the serum of mice were analyzed by CBA. Experimental groups: NC, negative control group; DSS, DSS group; LL-F, *L. lactis* MG1363 FnBPA+ group and LL-FT, *L. lactis* MG1363 FnBPA+ (pValac::*anti-TNFα*) group. Data are expressed as the means ± SEM from an experiment using 4-5 animals per group. Statistical analysis was performed using the Mann-Whitney test. *p < 0.05.


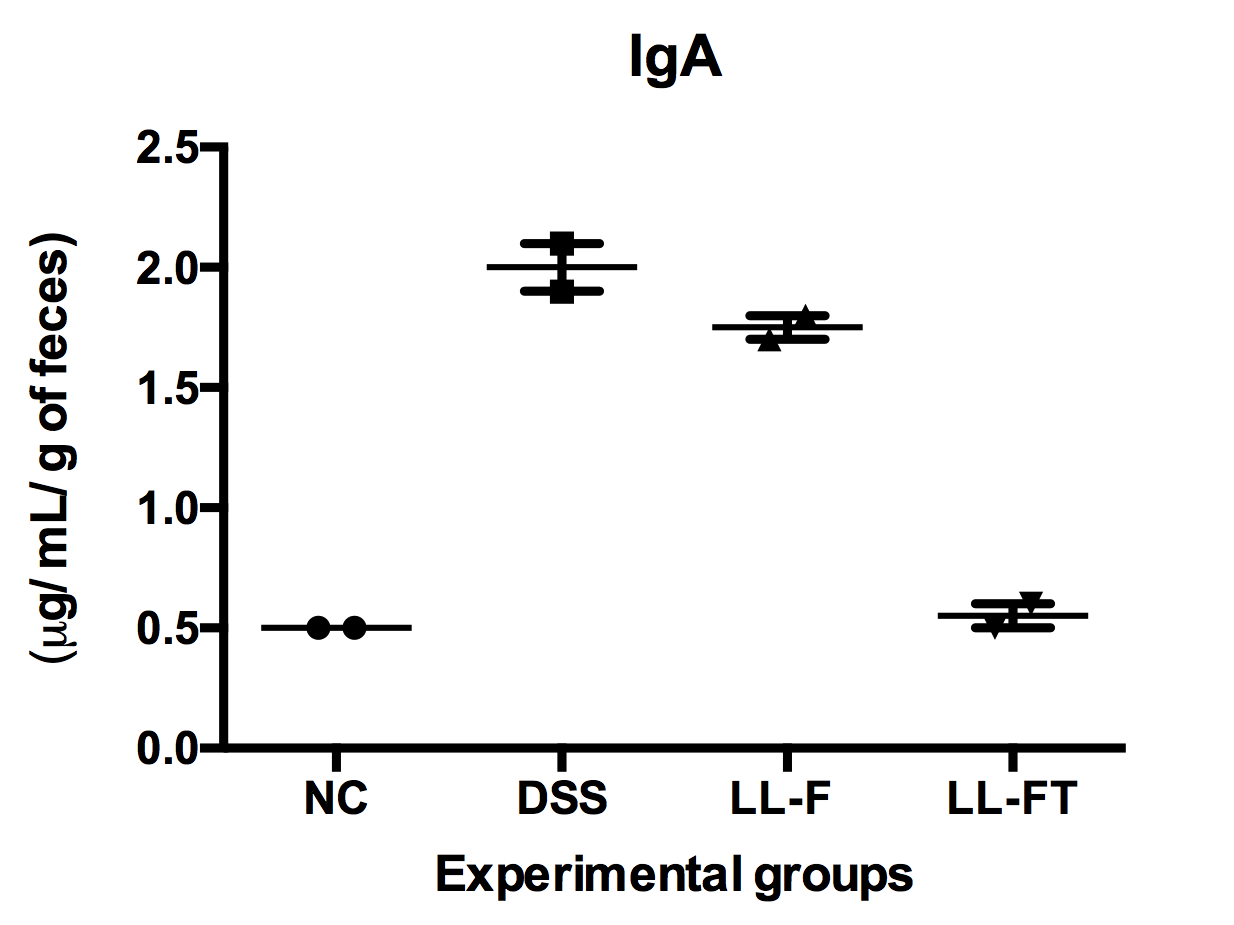


**Figure S4 Changes in fecal IgA after oral administration of LL-FT.** A pool of feces per group was collected 30 min before euthanasia, and the levels of fecal extract IgA were measured by ELISA. Experimental groups: NC, negative control group; DSS, DSS group; LL-F, *L. lactis* MG1363 FnBPA+ group and LL-FT, *L. lactis* MG1363 FnBPA+ (pValac::*anti-TNFα*) group. Data are expressed as the means ± SEM from an experiment using 4-5 animals per group. Statistical analysis was performed using the Mann-Whitney test.

# Additional Tables

**Table S1** Primer sequences used in qPCR assay.

| **Gene Target** | **Sequence 5’- 3’** |
| --- | --- |
| *tbx21* | FW TCAGGACTAGGCGAAGGAGA |
|  | RV GCCTTCGGTTTCCTTATCAA |
| *stat1* | FW TCACAGTGGTTCGAGCTTCAG |
|  | RV GCAAACGAGACATCATAGGCA |
| *rorc* | FW TGCAAGACTCATCGACAAGG |
|  | RV AGGGGATTCAACATCAGTGC |
| *arg1* | FW GTTCCCAGATGTACCAGGATTC |
|  | RV CGATGTCTTTGGCAGATATGC |
| *nos2* | FW CGAAACGCTTCACTTCCAA |
|  | RV TGAGCCTATATTGCTGTGGCT |
| *foxp3* | FW ACTCGCATGTTCGCCTACTT |
|  | RV AGGGATTGGAGCACTTGTTG |
| *Il6* | FW TAGTCCTTCCTACCCCAATTTCC |
|  | RV TTGGTCCTTAGCCACTCCTTC |
| *Il10* | FW CGCAGCTCTAGGAGCATGTG |
|  | RV GCTCTTACTGACTGGCATGAG |
| *tgfb* | FW CTGACGTCACTGGAGTTGTACGG |
|  | RV GGTTCATGTCATGGATGGTGC |
| *il1b* | FW CTGACGTCACTGGAGTTGTACGG |
|  | RV CAGCACGAGGCTTTTTTGTTG |
| *tnfa* | FW GTACCTTGTCTACTCCCAGGTTCTCT |
|  | RV GTGGGTGAGGAGCACGTAGTC |
| *muc3* | FW 5CGTGGTCAACTGCGAGAATGG |
|  | RV 5CGGCTCTATCTCTACGCTCTC |
| *il22* | FW GGTGACGACCAGAACATCCA |
|  | RV CCCAATCGCCTTGATCTCTC |
| *rps9* | FW CGCCAGAAGCTGGGTTTGT |
|  | RV CGAGACGCGACTTCTCGAA |
| *il17a* | FW TTTAACTCCCTTGGCGCAAAA |
|  | RV CTTTCCCTCCGCATTGACAC |

**Table S2** Histological Score.

| **Histological findings** | **Absent** | **Mild** | **Moderate** | **Severe** |
| --- | --- | --- | --- | --- |
| Mucosal infiltrate | 0 | 1 | 2 | 3 |
| Submucosal infiltrate |  |  |  |  |
| Muscle infiltrate |  |  |  |  |
| Serous infiltrate |  |  |  |  |
| Eosinophils |  |  |  |  |
| Muscle herniation |  |  |  |  |
| Inflammatory activity of glands |  |  |  |  |
| Abscesses of crypts |  |  |  |  |
| Erosion of mucosa |  |  |  |  |
| Mucosal ulceration without injury *Muscularis mucosae* |  |  |  |  |
| Long base ulcer |  |  |  |  |
| Muscle thickening |  |  |  |  |
| Depletion of goblet cells |  |  |  |  |
